# The Responsibility of the Profession in the Production of Opium Inebriety

**Published:** 1878-02

**Authors:** 


THE RESPONSIBILITY OF THE PROFESSION IN THE PRODUCTION OF OPIUM INEBRIETY—*Read before the American Association for the Cure of Inebriates.*—MR. PRESIDENT, GENTLEMEN: In accepting the invitation tendered me, to present a paper at this meeting, I have thought it might not be undesirable to direct attention to one phase of a certain topic which I have before presented to the profession—New York *Medical Record*, January 29th and December 9th, 1876—and therefore beg leave to offer briefly some remarks on "the responsibility of the profession in the production of opium inebriety."

That opium is largely used, and that its use during the last two or three decades has increased far in advance of its direct therapeutical need, are facts which no one rightly informed will question. That its habitual consumption will give rise, in a large proportion of cases, to a disease having symptoms as marked and specific as many others coming under professional cognizance, is another fact indisputable; and that this addition to our nosology has failed of receiving from the profession that attention it most deservedly demands, is as true as it is regretable.

Passing at once to the main point involved in this paper, what is the chief factor in the etiology of opium inebriety? Is it a matter of choice, that its victims indulge in it for any transient happiness it may furnish; any seeming oblivion it may give to disturbing emotions; any intellectual stimulation it may occasion; or, is it that, once ordered on professional authority, and continued a time indefinite, it causes such changes, mental and physical, as to engender a constant demand for its taking, and thus gives over its users to a servitude most deplorable? Diversity of opinion exists on this point, though the vast preponderance of testimony favors the statement that, in a large majority of instances, it is involuntary; that patients, unaware and unwarned of its ensnaring property, continue it, unsuspectingly, until the morbid condition becomes established. Dr. Parrish says: "Men take it, not for social enjoyment, but for a physical necessity." Dr. Van Deusen remarks: "For the primary induction of the habit, the stated physician is so commonly responsible, that the onus probandi in exculpation rests upon him, by all fair presumption." A late German writer asserts: "The original causes of this diseased condition, and of its extension, are the doctors themselves, who have accustomed patients to resort to the use of injections for the relief of painful affections of more or less short duration." Another foreign authority, after detailing several cases of this disease coming under his care, all having their inception in an opiate prescription, observes: "This leads me to say that I think there are many cases of chronic disease, or disease in which sleeplessness is a prominent symptom, in which opium is frequently prescribed without any thought of this danger supervening, and which has led me to believe and practice that it is advisable not to give opium continuously for any length of time in these cases, unless in painful and incurable diseases, as cancers, etc., especially if the patient is of a nervous temperament. There is no doubt that pain or sleeplessness would more frequently have to be endured, but other remedies might be substituted which would, in part, though not entirely, take its place. And if we consider that it would save some, more, perhaps, than many are aware of, from contracting such a habit, I think that at least it would be advisable to consider this danger, and bear in mind, when we are called upon to treat such cases and believe opium to be indicated—a danger which most readers well know, from their own experience, not to be a fanciful one."

After referring to the strictures sometimes passed regarding the medicinal employment of alcoholic stimulants, he continues: "But it is otherwise with those I have mentioned, who have not known the potency of this drug, at least personally, until it was professionally administered, and who, in all likelihood, would not have known it unless so ordered. And this is generally the rule. I think that the majority of the habitual opium eaters have, in the first instance, acquired the taste for it after having been ordered it by a medical man; and in view of this danger, I venture to say that this ought to be borne in mind when we administer opium in these cases, lest we might be the innocent cause of setting the spark to the fire that may only be extinguished with life." My personal knowledge, embracing the history of quite a number of instances, is fully confirmatory of these opinions, and I hazard little in expressing a belief that eighty per cent. of the cases of opium inebriety in this country may be traced, more or less directly, to opiate prescriptions. This assertion may seem somewhat extravagant, but I challenge proof to the contrary.

Now, the question naturally arises, why so large a degree of professional responsibility? The answer is to be found in the fact of our being a people peculiarly susceptible to the seductive influence of this drug, by virtue of our strongly-marked nervous temperament; the extensive prevalency of neurotic disorders, very largely of hereditary transmission, thus presenting the most favorable field for reaping a generous harvest of those ills of body and mind which follow habitual indulgence; and in the other great fact that practitioners have been too lavish in the expenditure of opiates for the relief of pain, insomnia and general nervous disturbance, without first fully informing themselves, by careful inquiry, respecting previous history on all points likely to conduce to a full knowledge of the risks incurred by a somewhat prolonged administration. Again, I believe a large share of causative influence may be found in the neglect of proper care on the part of physicians to see to it, thoroughly, when the strict therapeutical necessity for the use of opium has been fulfilled, that their patients utterly abandon it. I am unhesitatingly of opinion that a want of careful attention to this point has led to the full development of the disease in very many cases where otherwise it might have been throttled on the very threshold of its existence. This especial feature as to prophylaxis assuredly demands the most serious consideration.

But while insisting that the fraternity has a responsibility on this point which it is useless to try to evade, I am willing to admit that an auxiliary factor of great importance is to be found in the absolute freedom from anything like requisite restriction in the sale of opium and its preparations. Nothing prevents an apothecary from refilling an opiate prescription as often as it may be presented; nothing restrains him or any other from supplying whatever demand may be created for opium. This is radically wrong, and with proper limitations should be prohibited. Many cases of opium addiction would unquestionably never pass beyond a mere inception, were it not possible for those in whom the seeds of the disease have been planted to procure further supplies, and by thus continuing to "sow the wind," "reap a whirlwind" of misery, mental and physical, which it is difficult fully to conceive.

Without desiring to enter upon any discussion as to the comparative liability of various opiate preparations to beget inebriety, I wish to call attention to one phase of this question which has excited no little comment, and certainly merits consideration. I refer to the use of the hypodermic syringe. That the subcutaneous administration of morphia has contributed incalculably to the alleviation of human suffering cannot be doubted; but it has been asserted that increased facility for continued indulgence is thus afforded, with largely augmented liability of establishing disease. I have been informed that a prominent member of this association entertains this opinion, and condemns its use, claiming far greater danger of inebriety, than if morphia be given per orem. He contends that the unpleasant gastric effects and cerebral disturbance which it so often occasions when administered per via naturale, constitute a decided obstacle to its protracted employment, whereas the trifling pain of the puncture, the freedom from disagreeable sequelæ just cited, and the, to many, enjoyable tranquility of mind and body it produces, prove almost irresistible temptations to its continuance. How far this opinion is correct I cannot say, but it may safely be asserted that, from the extensive prevalency of this form of medication, many cases will be found of this character, and that the number will constantly increase, especially if the pernicious practice—which I am impelled to believe is quite extensively indulged in—of patients being professionally advised to purchase a syringe and personally employ it—be continued.

An interesting paper on "Opium Inebriety, and the Hypodermic Syringe," by Dr. McFarland, of Oxford, New York, read at the last meeting of the New York State Medical Society, pointed out the danger of this course, and advised strongly its discontinuance.

Having thus directed attention to a state of affairs largely existing, what is the remedy for the resultant evils?

First. A diminished prescribing of opiates, a lessened idea that they are the *sine qua non*, and the substitution of various anodynes, soporifics, and nervines, which, though they may somewhat imperfectly accomplish the object, as compared with opium, are free from that peculiar property which so often entails dire results.

Secondly. In all cases where their use seems indicated, careful inquiry by the attending physician, as to the neurotic status of the patient, from the standpoint of hereditary tendency, by thorough investigation of ancestral mortuary records, and a study of individual temperament, with the object of ascertaining the vulnerability of each case to the exceptionally ensnaring power of opium.

Thiidly. In every case, as limited an employment of opium, and as frequent using of substitutes, as circumstances will admit, interrupting an administration at short intervals, if possible, and thus lessening the chances of habituation.

Fourthly. Patients under the necessity of using opiates should be kept under careful and frequent observation, and immediately the direct need for their use has subsided, the attending physician should exercise fully his authority in compelling a peremptory abandonment. Pre-eminently so is this with the hypodermic syringe, and under no circumstances, except the case of painfully incurable disease—and even then it would be better to entrust its use to a friend—should a patient be permitted self-administration. Personal employment is fraught with danger, patients almost invariably using it to excess, and the practice of allowing it cannot be too severely condemned, or its discontinuance too strenuously insisted upon. More, medical attendants should deem it their duty to follow closely the course of patients for some time after opium has been abandoned, lest, from some pretext, its use be resumed clandestinely, and inebriety become established. More than one striking case of this kind, in which it was employed hypodermically, has fallen under my observation.

Careful observance of these precautions would, unquestionably, reduce largely to a minimum the ills resulting from the indiscreet employment of opium, and I trust the time is not far distant when this Society will deem it advisable to express a sentiment in accordance therewith, confident that, could it make itself heard and heeded, a vast amount of harm might be avoided.

Nor can I allow this opportunity to pass without asking whether the time has not arrived when it is the duty of this association to direct attention to the indiscriminate sale of opiates, and take measures looking to its restriction? Certain it is that, despite every professional precaution which may be observed, an avenue for continued indulgence is now open, which individual as well as public interests demands should be closed. Certain it is that, so long as no safeguard is afforded those in whom the earliest indications of this abnormal appetency are aroused, so long will we have constant accessions to the ranks, already surprisingly large, of opium habitues.

I am firmly of opinion that legislative enactment, prohibiting the refilling an opiate prescription, or the dispensing of opium in any form, without a positive order from the attending or other physician, would accomplish an immense amount of good, by protecting those who, otherwise, might become the pitiable possessor of an appetite making alike havoc of mind and body.

A precedent has been given us. During the past year our German brethren, with a far-reaching wisdom which will bear good fruit in the future, have secured restrictive legislation in this direction, and the good example thus presented we should make no delay in emulating. I commend this subject to the earnest consideration of this association. As the pioneer in every movement having at heart the protection of individuals and society against the ravages of inebriating agents, an ample field is here afforded for the exercise of those wise counsels and benificent works so potent for good in the past, and so pregnant with weal for the future. The prevention of disease ranks higher than its cure, and in the entire realm of prophylactic medicine I know of but few better opportunities for brilliant achievements than this.—*Medical and Surgical Reporter*.

---

Pepsine and pancreatine have been considered only temporarily beneficial, but their permanent benefits are well known to those familiar with them as remedies. The New York Pharmical Association proportions them with the supposed necessary acids for use.

